# Unusual presentation of a case of fetal hepatic mass: a case report

**DOI:** 10.1186/s12884-023-05626-1

**Published:** 2023-04-26

**Authors:** Dongmei Liu, Jiali Yu, Yang Yang, Minzhi Ouyang, Ming Zhang, Shi Zeng, Ganqiong Xu

**Affiliations:** 1 Department of Ultrasound Diagnosis, The Second Xiangya Hospital, Central South University, 139 Renmin Road (M), Changsha, 410011 Hunan China; 2 Research Center of Ultrasound Diagnosis, The Second Xiangya Hospital, Central South University, Changsha, 410011 Hunan China; 3 Clinical Research Center for Medical Imaging in Hunan Province, Changsha, 410011 Hunan China

**Keywords:** Prenatal diagnosis, Ultrasound, Congenital hepatic tumor, Congenital hepatic hemangioma, Case report

## Abstract

**Background:**

Giant hepatic hemangiomas are rare and can cause serious complications that contribute to a high risk of perinatal mortality. The purpose of this article is to review the prenatal imaging features, treatment, pathology, and prognosis of an atypical fetal giant hepatic hemangioma and to discuss the differential diagnosis of fetal hepatic masses.

**Case presentation:**

A gravida 9, para 0 woman at 32 gestational weeks came to our institution for prenatal ultrasound diagnosis. A complex, heterogeneous hepatic mass measuring 5.2 × 4.1 × 3.7 cm was discovered in the fetus using conventional two-dimensional ultrasound. The mass was solid and had both a high peak systolic velocity (PSV) of the feeding artery and intratumoral venous flow. Fetal magnetic resonance imaging (MRI) revealed a clear, hypointense T1-W and hyperintense T2-W solid hepatic mass. Prenatal diagnosis was very difficult due to the overlap of benign and malignant imaging features on prenatal ultrasound and MRI. Even postnatally, neither contrast-enhanced MRI nor contrast-enhanced computed tomography (CT) was useful in accurately diagnosing this hepatic mass. Due to persistently elevated Alpha-fetoprotein (AFP), a laparotomy was performed. Histopathological examination of the mass showed atypical features such as hepatic sinus dilation, hyperemia, and hepatic chordal hyperplasia. The patient was ultimately diagnosed with a giant hemangioma, and the prognosis was satisfactory.

**Conclusions:**

When a hepatic vascular mass is found in a third trimester fetus a hemangioma should be considered as a possible diagnosis. However, prenatal diagnosis of fetal hepatic hemangiomas can be challenging due to atypical histopathological findings. Imaging and histopathological assays can provide useful information for the diagnosis and treatment of fetal hepatic masses.

## Background

Congenital hepatic tumors are rare. The three main types of primary congenital hepatic tumors are hemangioma (60.3%), mesenchymal hamartoma (23.2%) and hepatoblastoma (16.5%) [1]. Among these, congenital hepatic hemangioma (CHH) is typically a solitary tumor of mesenchymal origin, characterized by rapid initial growth and frequent spontaneous regression. A hepatic hemangioma larger than 40 mm in diameter is clinically defined as a giant hepatic hemangioma [2]. Due to the scarcity of reports of fetal giant hepatic hemangiomas in the literature, the true incidence of giant hepatic hemangioma is unknown [3]. Although fetal hepatic hemangioma is a benign tumor, its prognosis is variable and depends on tumor size, growth rate, and arteriovenous fistula shunting within the tumor. Clinical manifestations vary from asymptomatic to life-threatening. Giant hepatic hemangiomas can cause serious complications such as arteriovenous fistula shunts and tumor rupture [4]. The presence of arteriovenous fistula shunts can lead to high-output heart failure and hydrops. Furthermore, fetal anemia can occur as a result of thrombocytopenia in consumptive coagulopathy (Kasabach-Merritt syndrome) [5, 6]. These complications are associated with perinatal mortality rates as high as 70% to 90% [2]. Therefore, the prenatal evaluation of fetal intrauterine conditions is particularly important. This article reports a case of an atypical giant hepatic hemangioma and analyzes the cause of its unusual presentation.

## Case presentation

**Fig. 1:**
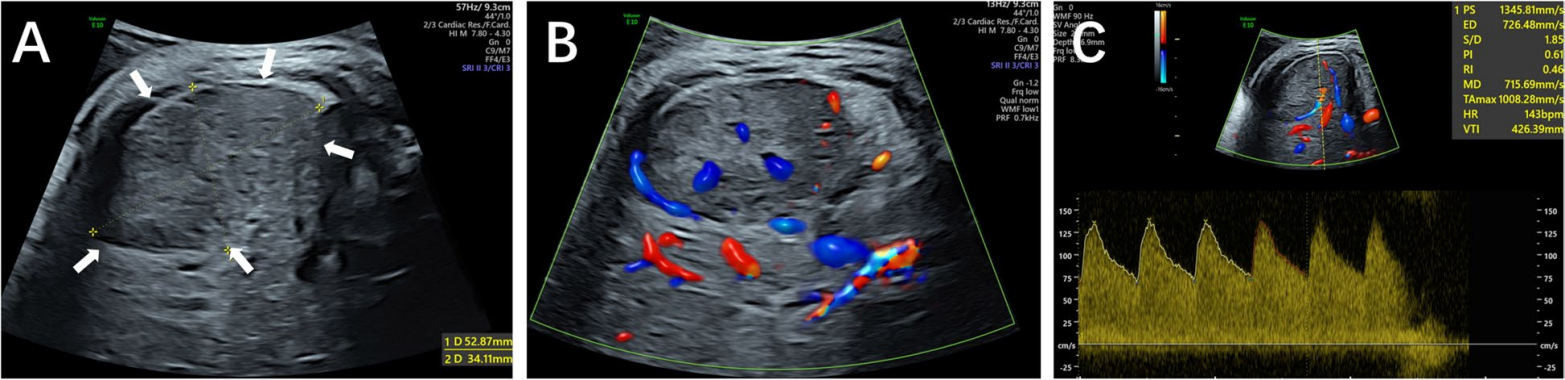
Prenatal ultrasonography imaging showed a voluminous, solid and heterogeneous hepatic mass. **A** Two-dimensional ultrasonography revealed a well-circumscribed, hypoechoic, and predominantly solid mass of approximately 5.3 × 4.0 × 3.4 cm in the right lobe of the fetal liver (arrow). **B** Color doppler showed a hypoechoic mass in the right liver of the fetus with abundant strip blood and peripheral blood. **C** Pulse doppler showed that the blood flow signal of the fetal right lobe mass displayed an arterial spectrum with a peak systolic velocity of the feeding artery of 1.35 m/s and a resistance index of 0.46

**Fig. 2:**
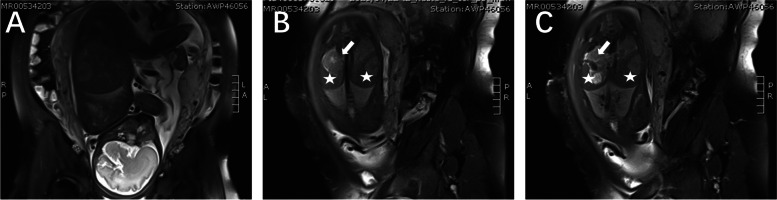
Prenatal MRI features of the fetal hepatic mass. **A**-**C** A large hepatic mass (arrow) with high signal intensity was seen in a sagittal T2-W image compared to the normal liver (star)

A 34-year-old Chinese woman (gravida 9, para 0, abortion 8) underwent first- and second-trimester routine ultrasound screening that did not reveal any pathological findings. However, prenatal sonography at 32 gestational weeks revealed a well-defined, solid 2.4 × 2.2 × 2.0 cm-sized mass in the right hepatic lobe that was peripherally hypoechoic and centrally hyperechoic. During fetal breathing movement, the mass showed synchronized movement with the liver. Color Doppler imaging of the lesion revealed prominent vascularity both within the lesion and surrounding it. The peak systolic velocity (PSV) of the feeding artery was 1.35 m/s, and the resistance index (RI) was 0.46 (Fig. 1). No arteriovenous fistula was observed in this lesion. There were no signs of fetal heart failure, hydrops, or anemia as the PSV of blood flow in the middle cerebral artery was 46 cm/s, and the blood flow spectrum of the umbilical artery and ductus venosus were normal. Fetal magnetic resonance imaging (MRI) demonstrated a clear, hypointense T1-W and hyperintense T2-W solid hepatic mass originating in the right hepatic lobe (Fig. 2). The parents received thorough counseling concerning the mass and its uncertain prognosis. Considering their poor obstetric history, the parents decided to continue the pregnancy. Serial ultrasound assessments revealed regular growth of the lesion in the following weeks, as shown in Table 1.

**Table1:** Follow-up results of clinical and imaging features of the fetal hepatic mass

Diagnosis	Tumor size (cm)	HC (cm)	V(cm^3^)	THCR	Echo	EF	Comp	CTR	CT/MRI	AFP (ng/ml)
Prenatal 32 weeks	2.4 × 2.2 × 2.0	28.3	5.49	0.19	periphery hypoechoic and central hyperechoic	No	Unclear	0.30	-	-
Prenatal 34 weeks + 1 day	4.0 × 3.2 × 3.0	30.7	19.97	0.65	SAA	No	Unclear	0.31	MR high T2W, low T1W	-
Prenatal 35 weeks + 5 days	5.3 × 4.0 × 3.4	32.0	38.58	1.20	SAA	No	Unclear	0.20	-	-
Prenatal 36 weeks + 1 day	5.2 × 4.1 × 3.7	32.2	41.02	1.27	SAA	No	Unclear	0.30	-	-
Postnatal 0 day	5.5 × 4.6 × 3.8	34.9	49.99	1.43	hyperechoic	No	Clear	0.31	-	> 600
Postnatal 7 days	5.8 × 4.4 × 3.7	35.7	49.1	1.38	SAA	No	Clear	0.30	-	259
Postnatal 14 days	5.8 × 4.4 × 3.7	36.1	49.1	1.36	SAA	calcification	Clear	0.29	-	322
Postnatal 1 month	5.5 × 4.5 × 3.7	37.1	47.62	1.28	SAA	calcification	Clear	0.31	-	351
Postnatal 1 month + 26 days	5.6 × 4.7 × 3.3	38.2	45.17	1.18	SAA	calcification	Clear	0.31	CT Peripheral rim enhancement	2490
Postnatal 2 months + 8 days	-	-	-	-	-	-	-	-	MR Peripheral rim enhancement	1341
Postnatal 3 months (postoperative)	-	-	-	-	-	-	-	-	-	286.42
Postnatal 5 months	-	-	-	-	-	-	-	-	-	117.06
Postnatal 10 months	-	-	-	-	-	-	-	-	-	7.39

*Abbreviation*: *HC* Head Circumference, *V* Volume, *THCR* Tumor Head circumference ratio, *Echo* Echogenicity, *EF* Echogenic Foci, *Comp* Compressibility, *CTR* cardiothoracic ratio, *CT/MR* Computer Tomography/Magnetic Resonance Imaging, *AFP* Alpha-fetoprotein, *SAA* Same as above

**Fig. 3:**
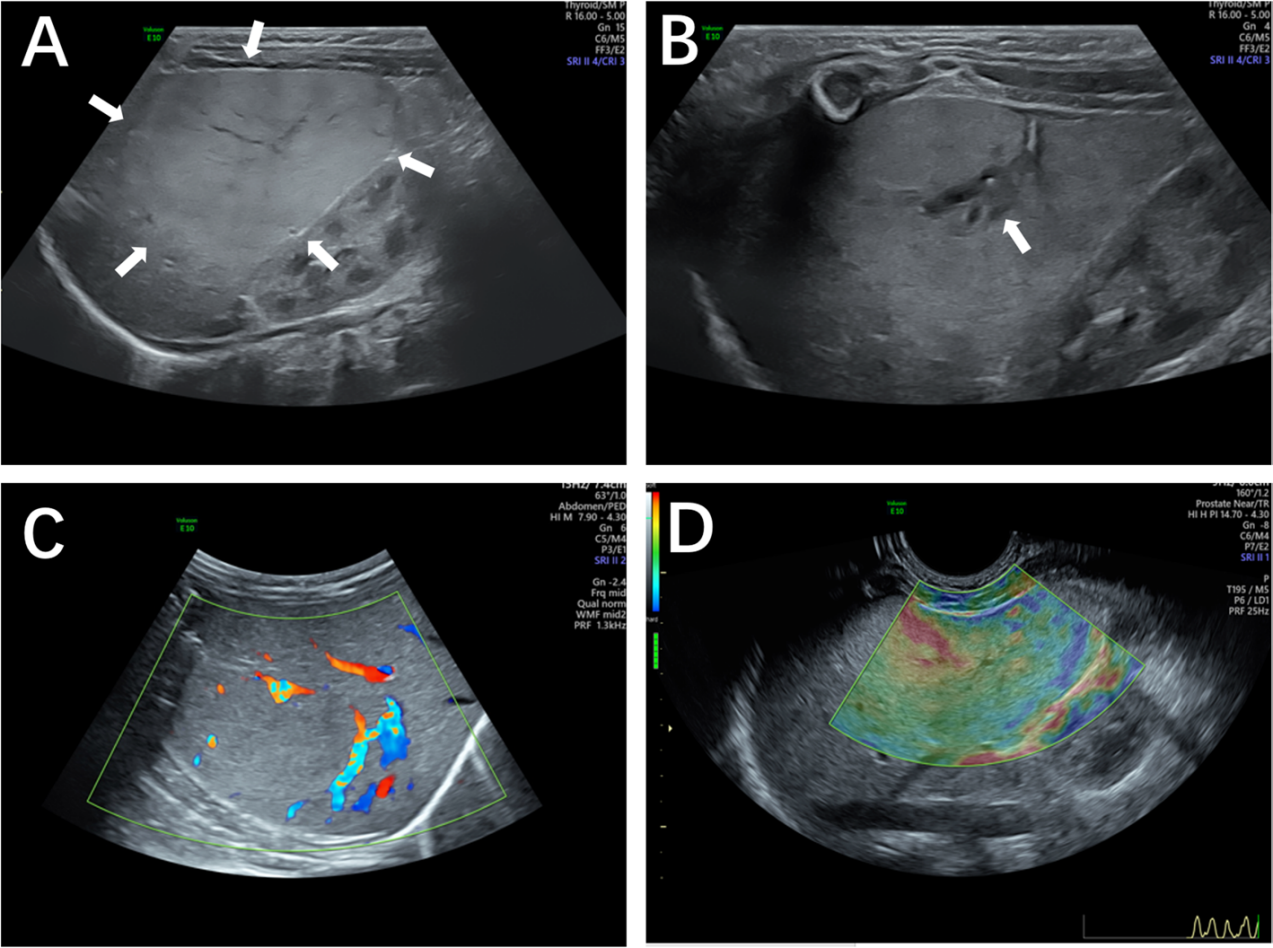
Postnatal ultrasound image of a right lobe hepatic mass. **A**-**B** Two-dimensional ultrasound showed a solid hyperechoic mass of approximately 5.5 × 4.5 × 3.7 cm (**A** arrow) in the infant's right liver, with clear borders, an irregular shape, and a 1.4 × 0.7 cm sheet-like anechoic area with strong light spots (**B** arrow). **C** Color doppler showed abundant blood flow signals around and inside the hyperechoic mass of the right liver, and the arterial and venous spectra could be detected. **D** Elastography revealed a hyperechoic right hepatic mass with low stiffness and compressibility

**Fig. 4:**
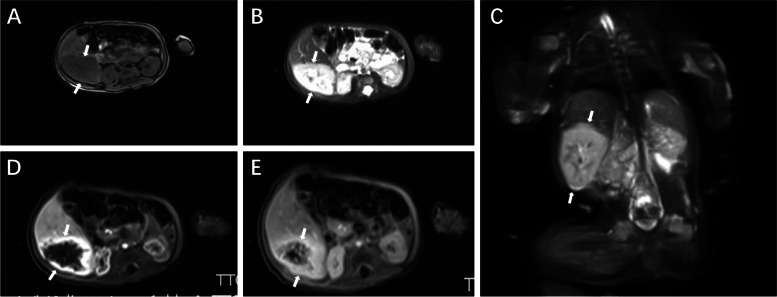
Postnatal MRI showed a large mass in the right lobe of the liver. **A**-**C** The transverse and coronal views of MRI showed a large mass in the right lobe of the liver, approximately 6.0 × 4.1 cm in size, low T1-W and high T2-W signals without signs of a false envelope, and adjacent compression of the right kidney. **D**-**E** Uneven enhancement of the edge of the mass was observed in the arterial phase of enhanced scanning. With the delay in scanning time, the enhancement area gradually widened and expanded to the center, but was not completely filled

A female infant weighing 3,140 g was spontaneously delivered vaginally at 36 weeks and 5 days, with Apgar scores of 9 at 1 min and 10 at 5 min. Postnatal sonographic examination confirmed the presence of a hepatic mass measuring 5.5 × 4.6 × 3.8 cm (Fig. 3). There were multiple echogenic foci with acoustic shadowing within the mass, suggesting calcifications. Elastography revealed the mass with low stiffness and compressibility. Both postnatal MRI and computed tomography (CT) were indicated to further characterize this lesion, which showed a heterogeneous early peripheral enhancement of the mass after intravenous contrast injection (Fig. 4). At 2 months and 8 days, the infant presented with mild anemia (hemoglobin 89 g/L) and normal coagulation. In addition, initial blood tests revealed normal liver function. However, serum Alpha-fetoprotein (AFP) levels were higher than normal (normal reference value < 20 ng/ml [7]) and fluctuated (Table 1).

**Fig. 5:**
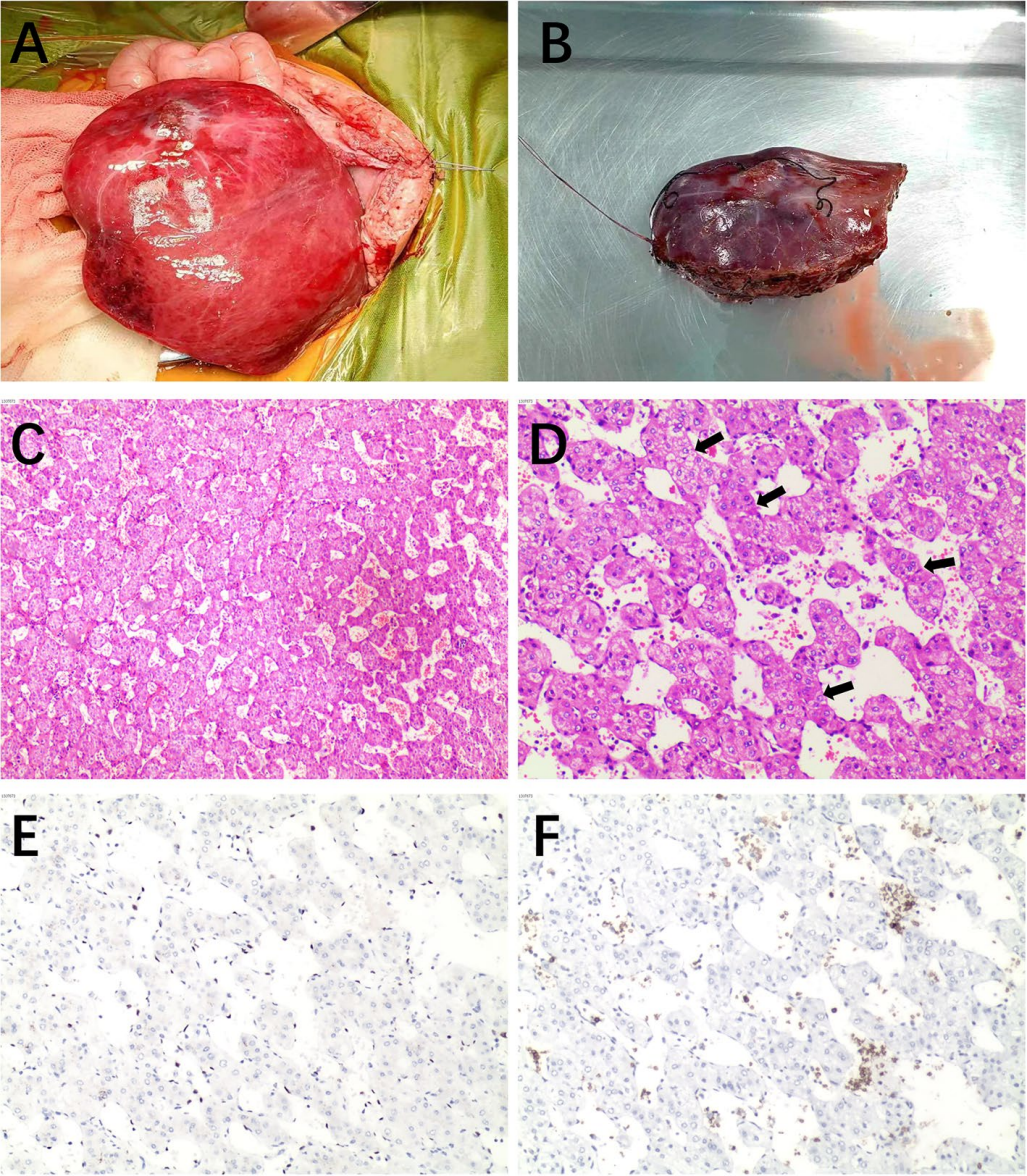
Postoperative gross specimens, pathology and immunohistochemistry. **A**-**B** In gross tissue, a hepatic mass with approximately 6.5 × 3.3 × 4.0 cm in size can be seen with the naked eye. The cross section was solid gray‒white and reddish‒soft. A 3.5 × 3.0 cm gray‒brown gray‒yellow area was present in the center. **C **Hepatic congenital hemangioma H&E 40 × staining showed dilated sinus congestion. **D** H&E 100 × : Lobules of different sizes are seen, mostly small, thin-walled blood vessel gaps, surrounded by more blood vessels filled with red blood cells and hepatic cord hyperplasia (arrow), without cytonuclear atypia. **E** ERG ( +) 100 × : Erythroblast transformation-specific-related gene expression of endothelial cells. **F** GLUT-1 (-) 100 × : No GLUT1 expression was observed in endothelial cells

Ultimately, because imaging and laboratory studies could not conclusively rule out a malignancy, under the advice of a pediatrician, a laparotomy was performed postnatally at 2 months and 10 days, and the mass was resected. The mass was dark red and contained mixed areas of necrosis. Histopathological examination of the mass showed hepatic sinusoidal dilatation and congestion, and hepatic cord hyperplasia (2–3 layers of cells), but no obvious cell atypia. Combined with immunohistochemical results showing CD34 ( +), CD31 ( +), SMA (-), D2-40 (-), ERG ( +), and Glut-1 (-), a diagnosis of congenital hemangioma was ultimately considered (Fig. 5). The patient had a good prognosis after one year of follow-up.

## Discussion and conclusion

Hepatic tumors are rare and account for approximately 5% of all tumors in the fetus and newborn [8]. The most frequently occurring are benign hepatic hemangiomas. Although benign in pathology, extensive hemangiomas can cause fetal anemia, fetal heart failure, and even intrauterine death [4, 5]. When a fetal hepatic mass is discovered, the nature and complications of the tumor need to be clarified. In this case report, the unusual ultrasound presentation made it difficult for us to accurately determine the nature of this tumor. We therefore focused on the causes of overlapping imaging features and explored whether there are specific ultrasound imaging features that can differentially diagnose hepatic masses.

### Analyzing the causes of unusual ultrasound presentations

This mass combined benign and malignant ultrasonic features. First, in grayscale ultrasound features, it mainly presented as a solid mass without the typical honeycomb-like changes of a hemangioma. We analyzed the imaging observation of a solid mass caused by hepatic cord hyperplasia.This event may have given rise to the dense structure of the mass rather than the “honeycomb” or “grid-like” appearance of typical hemangiomas [9]. Second, in Color Doppler ultrasound, this mass had both a high PSV of the feeding artery and intratumoral venous flow. A PSV of the feeding artery higher than 0.40 m/s typically indicates a malignant hepatic tumor rather than a hemangioma [10], which intratumoral venous flow is characteristic of a benign tumor [10, 11]. Conventional typical hemangiomas do not usually show hyperplasia of the hepatic cord [12]. The hyperplasia of the hepatic cord in this atypical hemangioma (which suggests active growth) may have contributed to the increased flow velocity of the feeding artery.

### Differential diagnosis of fetal hepatic masses

We encountered unusual presentations that made it difficult to determine the nature of this mass. The differential diagnosis of fetal hepatic masses was particularly important in this case and included hemangiomas, hepatoblastomas, mesenchymal hamartomas, and metastatic malignant tumors. Fetal hepatoblastoma is a malignant hepatic tumor that arises during in the fetal period, and similarly to hepatic hemangioma, is often detected in late pregnancy. It usually appears as a large, lobulated, and heterogeneous solid tumor [13]. The key investigation is that hepatoblastoma can usually lead to an increase in AFP levels. In the first few months of life in infants, AFP levels are normally elevated and can easily mask elevated AFP levels caused by malignant lesions [14]. However, in this case, the fluctuating increase in AFP levels in this infant indicated the possibility of a malignant tumor, which was one reason why this infant underwent surgical removal of the mass as soon as possible. According to Belinda et al., giant hepatic hemangiomas can be heterogeneous and may have a central necrotic hypoechoic area inside [9]. However, when a solid mass of hepatoblastoma undergoes necrotic liquefaction, it can also present as a central hypoechoic mass. In such a situation, it is difficult to distinguish between them. In addition, mesenchymal hamartomas may be more commonly multicystic with echogenicity and low vascularity [15]. Hepatic metastatic malignant tumors may have a different echogenic pattern and blood supply characteristics that correlate with the primary lesion.

Based on a review of the literature, we find that ultrasound examination can be used to identify several types of fetal hepatic tumors and characterize their location, shape, boundary, composition, vascular distribution, and compressibility as shown in Table 2. To the best of our knowledge, atypical imaging manifestations of giant hepatic hemangioma have not yet been reported. This study may therefore be useful for the prenatal diagnosis, detection, and guiding treatment of hepatic masses.

**Table 2:** Differential diagnosis of fetal hepatic tumors by prenatal ultrasound [11, 15–18]

	Incidence	Location	TB	Shape	Composition	Echo	CD	PSV	RI	Others	Comp
hemangioma	60.3%	in either or both lobes	well-defined	regular	solid lesions with hypervascularization	hypo-, iso- or hyperechogenic	high vascularity	low PSV(< 0.4 m /s)	low RI (< 0.7)	fine granular calcifications	Yes
hepatoblastoma	16.5%	more than 60% of the cases arise from the right lobe	well or ill-defined	irregular	solid with poor vascularization	hypoechoic with foci of hemorrhage and necrosis inside	less vascularity	high PSV (≥ 0.4 m /s)	high RI (> 0.7)	coarse and dense calcifications	No
mesenchymal hamartoma	23.2%	anywhere in the liver	well-defined	regular	multicystic, multilocular	anechoic	low vascularity	**-**	**-**	calcifications	NA
metastatic malignant tumors	unknown	anywhere in the liver	ill-defined	irregular	multi-focal lesions	hypoechoic	high vascularity	high PSV (≥ 0.4 m /s)	high RI (> 0.7)	solid masses with a bull’s-eye configuration	NA

*Abbreviation*: *TB* Tumor borders, *CD* Color Doppler, *PSV* Peak systolic velocity, *RI* Resistance index, *Comp* Compressibility, *NA* Not assessed

In conclusion, the pathophysiological changes of various hepatic masses often present with overlapping imaging features during prenatal imaging. Prenatal ultrasound is a better method for detecting fetal hepatic masses. However, it remains difficult to identify the type of mass based on ultrasound alone. A comprehensive diagnosis should be made based on medical history combined with imaging and clinical data. Finally, ultrasound is a convenient method for follow-up and can guide clinically actionable and effective interventions.

## Data Availability

All data generated or analyzed during this study are included in this published article.

## Abbreviations

US: Ultrasound
CT: Computer Tomography
MRI: Magnetic Resonance image
AFP: Alpha-fetoprotein
CHH: Congenital hepatic hemangioma
PSV: Peak systolic velocity
RI: Resistance index
